# Deep ocean hydrographic variability estimated from distributed geodetic sensor arrays off northern Chile

**DOI:** 10.1038/s41598-024-61929-z

**Published:** 2024-05-15

**Authors:** Anna Jegen, Dietrich Lange, Johannes Karstensen, Oscar Pizarro, Heidrun Kopp

**Affiliations:** 1 GEOMAR Helmholtz-Zentrum für Ozeanforschung Kiel, Kiel, Germany; 2 Universidad de Concepción, Barrio Universitario s/n, Concepción, Chile

**Keywords:** Physical oceanography, Geophysics

## Abstract

Observations of spatio-temporal variability of the deep ocean are rare and little is known about occurrence of deep ocean mesoscale dynamics. Here, we make use of 2.5 years of time series data from three distributed sensor arrays, which acquired high-resolution temperature, pressure and sound speed data of the bottom layer offshore northern Chile. Estimating salinity and density from the direct observations enable access to the full spectrum of hydrographic variability from a multi-hourly to annual time scale and with average inter-station distances of less than 1 km. Analyses revealed interannual warming over the continental slope of 0.002 °C yr^−1^–0.003 °C yr^−1^, and could trace periodic hydrographic anomalies, likely related to coastal-trapped waves, as far as to the lower continental slope. A concurrent change in the shape of the warm anomalies and the rate of deep-sea warming that occurs with the crossing of the deep-sea trench suggests that the abyssal part of the eastern boundary current system off Chile does not extend past the deep sea trench. Furthermore, the comparison of anomaly timing and shape in between stations implies southwards flow over the mid to lower continental slope, centred closer to the trench.

## Introduction

With a mean depth of about 3700 m, the majority of the global ocean volume lies below 2000 m and thus below the depth of operational observation systems like the Argo array^1^. Despite being an important heat and carbon sink, that hosts highly specialised ecosystems, energetic currents and resources, the deep ocean remains chronically undersampled and hence understudied^2^. One main reason why our knowledge of the deep ocean is restricted is that systematic deep ocean observations are typically limited to address either synoptic spatial resolving observations (ship surveys) or single point time resolving (moored sensors) observations^3^. However, dedicated experiments using Lagrangian floats have revealed that eddy kinetic energy in the deep ocean can show regional highs^4,5^. Similarly, high resolution (a couple of km’s grid size) numerical model simulations have reproduced meso- and even submesoscale dynamics in the deep ocean^6,7^, which were often related to current-topography interactions. The interaction of period flow from tides with rough topography has recently been linked to deep ocean mixing and is understood to be key for maintaining the large-scale ocean stratification and circulation^8^. Nevertheless, our understanding of spatial and temporal variability of the deep ocean is fragmentary and patchy at best, due to a significant lack of in situ observations. In recent years, a growing number of offshore geodesy experiments has targeted the deep ocean in order to evaluate marine geohazards by assessing tectonic deformation through strain measurements^9–11^. Because of the strong frequency-dependent damping of electromagnetic waves and the low resolution of gravity models, many of these experiments rely on acoustic ranging as means to measure seafloor strain. The resolution of acoustic-ranging based geodesy methods is limited predominantly by the accuracy of the ambient sound speed information and thus dependent on the correct representation of temporal variations in temperature, pressure, and salinity^12^. Therefore, despite having contrasting objectives, oceanographic and offshore geodetic experiments show great overlap in the measured parameter space, evoking the possibility of cross-beneficial studies^13^.

**Figure 1:**
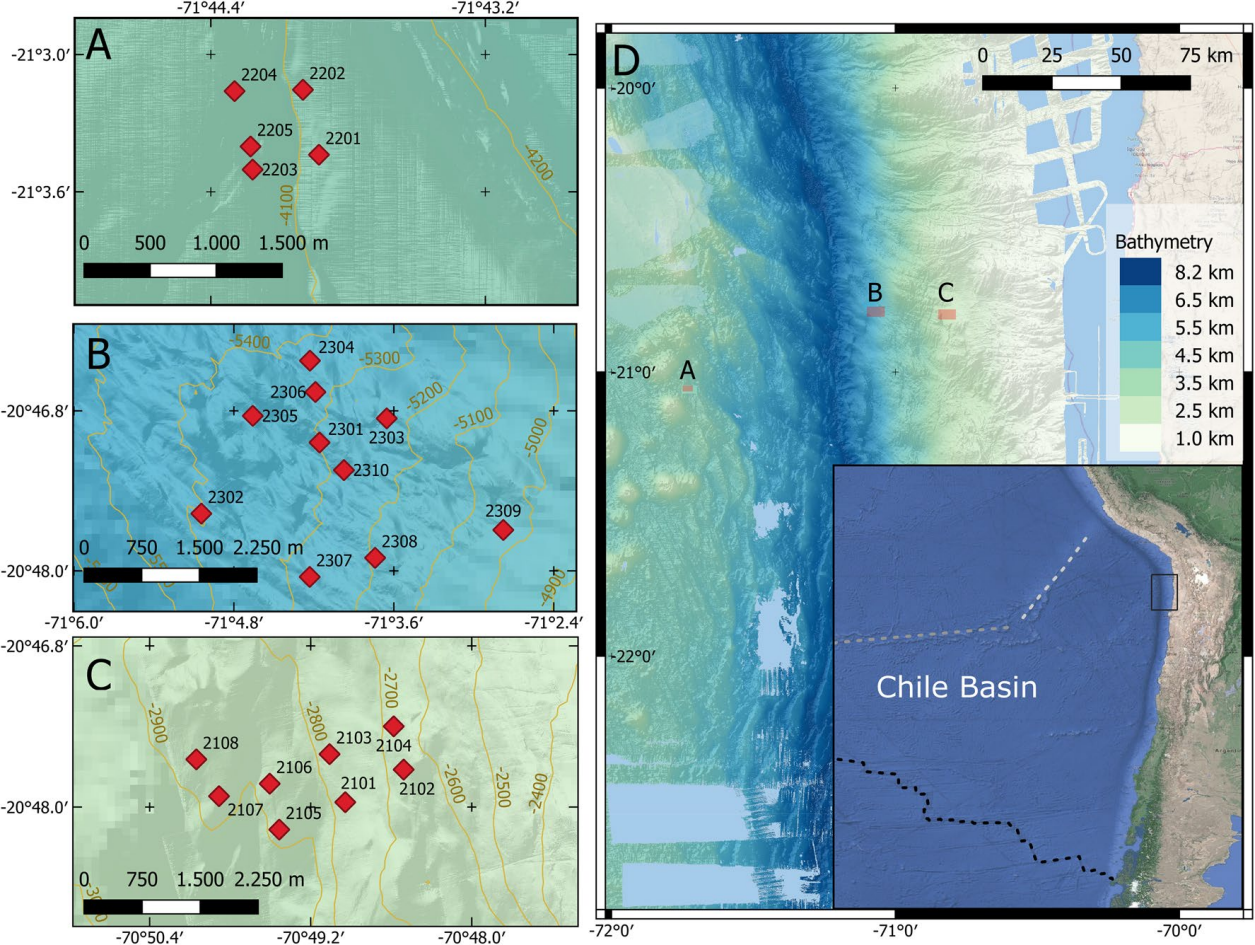
Overview map. Location of the 23 seafloor stations measuring temperature, pressure and sound speed sensors offshore Chile. Panels A, B and C show the station locations on the oceanic plate, lower and mid continental slope arrays, respectively. Topography contour lines (100 m intervals) from autonomous underwater vehicle (AUV) bathymetry are shown in dark green in panels (**A**–**C**). Red diamonds mark the locations of the stations. The overview map located within panel (**D**) was created with previously published data^15^ and shows the Chile Basin, bounded by the Chile Rise (dashed black line), the Sala y Gomez Ridge (dashed grey line) and the Nazca Ridge (dashed white line). The black rectangle indicates the location of the multi-array experiment.

The high-resolution (sampling frequencies of 90 min as well as 160 min, respectively) time series of deep in-situ temperature, pressure and sound speed were acquired at three representative locations across the northern Chilean deep-sea trench (Fig. 1) and are, to the authors knowledge, the longest continuous observation of bottom layer temperature variability in the eastern Pacific Ocean. The data were compared to published oceanographic reference data from the GLODAPv2 climatology^14^ and were originally analysed in order to refine follow up geodetic analyses. While the high level of coherence found both in between sensors and the climatology suggested a high level of precision, absolute temperature offsets both in between stations and to the climatology suggest some sensor bias related to the absolute temperature values. Because of this, later analyses of the temperature data are restricted to relative temperature changes, in which time series are understood as cold and warm anomalies that oscillate around a regional mean value derived from GLODAPv2. The objective of the study is to characterise and quantify spatial and temporal hydrographic variability, to determine whether and to which extent, prominent dynamical features of the deep ocean, such as tidal currents and barotropic topographic waves can be observed in geodetic data.

## Results

### Temporal fluctuations

**Figure 2:**
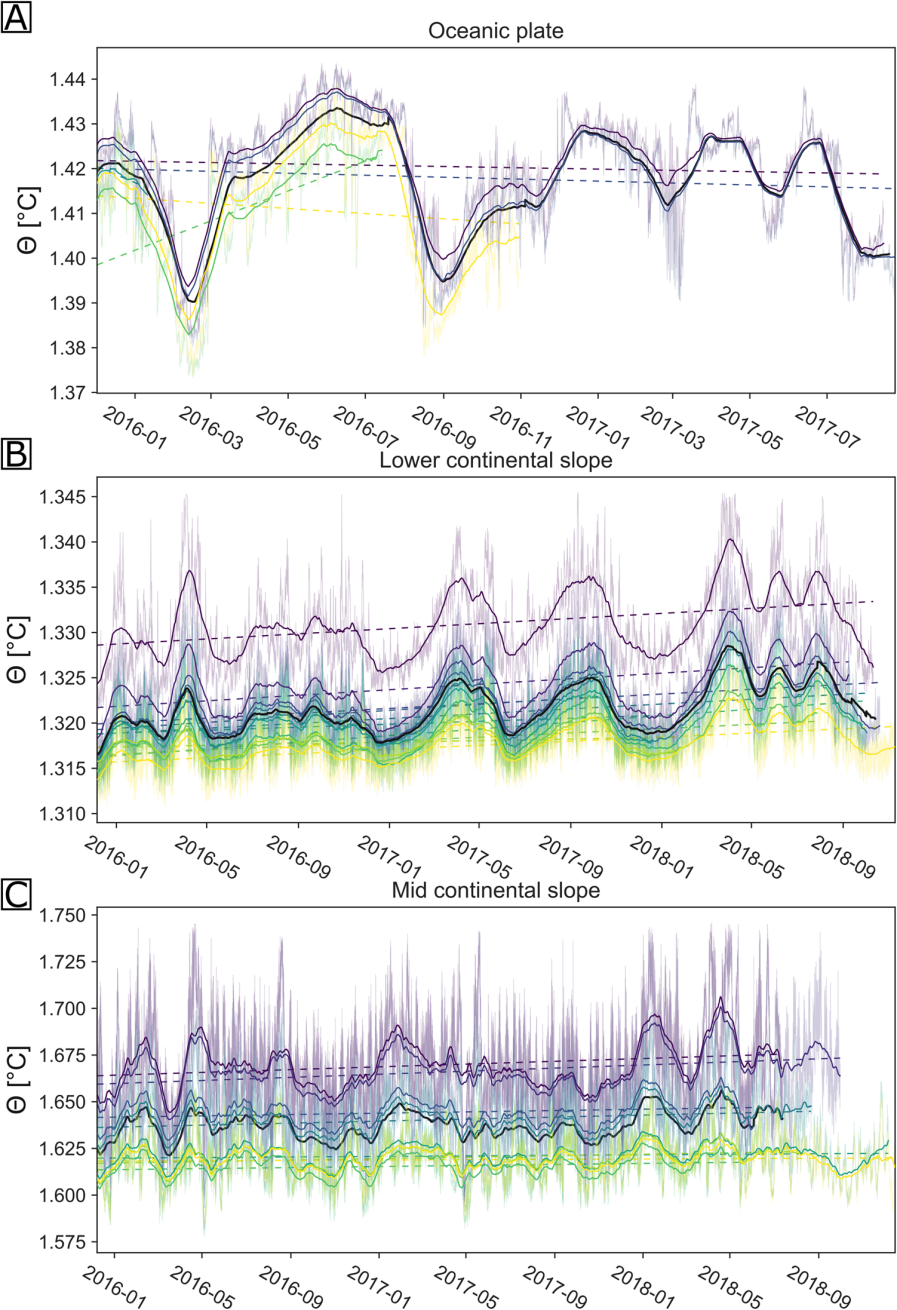
Time series of temperature data. Thin lines show the original data and solid lines the monthly running averages that were calculated with a minimum period of 7 days. The monthly average of each station is shown colour-coded according to the relative depth of the station and the average of all the array’s temperature records was added in black. Panel (**A**) shows the oceanic plate site, panel (**B**) the lower continental slope array and panel (**C**) the mid continental slope array. The interannual temperature trend of each station, determined via linear regression of each time series is marked by the dotted lines, coloured in accordance with the station’s measured potential temperature.

The average potential temperatures recorded at the oceanic plate site, the mid and lower continental slope sites (Fig. 1) varies around 1.42 °C, 1.32 °C and 1.64 °C over the course of the experiment, and shows the expected decrease of temperature with increasing water depth (Table 1). Temporal temperature oscillations are pronounced (Fig. 2) and operate with a wide range of frequencies in the data of all three sensor arrays.

**Table 1:** Observed variability and mean of the analysed data.

	Oceanic plate	Lower continental slope	Mid continental slope
Water depth covered by array	4107 m to 4030 m	5028 m to 5374 m	2635 m to 2880 m
Potential temperature range (Mean)	1.373 °C to 1.443 °C (1.42 °C)	1.311 °C to 1.37 °C (1.32 °C)	1.579 °C to 1.741 °C (1.64 °C)
Mean salinity	34.692 PSU	34.692 PSU	34.692 PSU
Mean potential density	1027.74 kg m^−3^	1027.75 kgm^−3^	1027.69 kgm^−3^
Mean potential temperature trend	0.018°Cyr^−1^	0.002°Cyr^−1^	0.003°Cyr^−1^

Overview of potential temperature, determined interannual trend, salinity and derived potential density range at each array after calibration.

**Figure 3:**
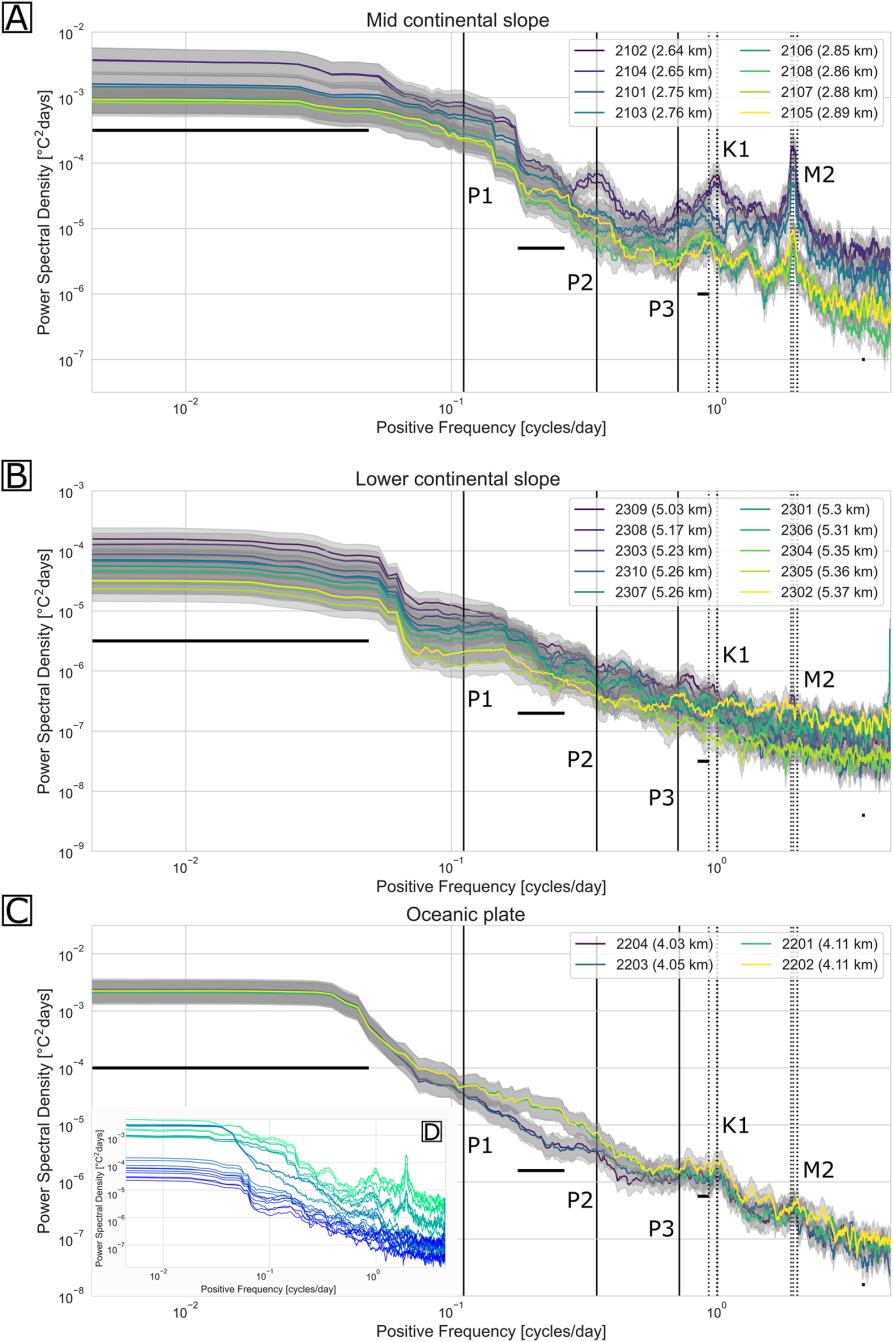
Spectral estimates of the temperature records. Spectral estimate of all acquired temperature series for the stations of the three arrays (**A**–**C**) estimated by the multitaper method using a time-bandwidth product of 10. The spectral estimates are coloured according to the station’s relative depth (shallow (purple) to deep (yellow)). The upper and lower 95 percent confidence levels of each spectral estimate are indicated in grey. The four horizontal lines represent the effective degree of smoothing that was applied during the multitaper method at the corresponding frequencies. Tidal frequencies are marked by dashed lines, including (from left to right) three diurnal tidal frequencies (marked K1) and four semidiurnal tidal frequencies (marked M2). The inertial frequency (marked P3), a 0.3–0.4 cycles day^-1^ frequency (marked P2) and a 0.1–0.125 cycles day^-1^ frequency (marked P1), which is likely related to coastal-trapped waves, could be observed in some of the spectral estimates and were marked by the solid vertical lines. The spectral estimates of all three sites are compared in panel D, also colour-coded in accordance to their relative depth (shallow (green) to deep (blue)).

In order to quantitatively analyse the periodicity of the temperature fluctuations, the spectral estimates of all temperature series were calculated using the multitaper method. Figure 3 shows the spectral estimate of all stations. No spectrum is shown for the data of station 2205, because of the short record length (~ 1 month) and our aspiration to ensure comparability in between the spectral estimates by having uniform Nyquist and Rayleigh frequencies. The overall energy level of the spectral estimates decreases with increasing water depth, which is why the temperature fluctuations are the largest at the shallowest location on the mid continental slope (Fig. 3C), second largest at the oceanic plate site (Fig. 3A) and smallest at the deepest site on the lower continental slope (Fig. 3B).

Given the decrease in temperature gradient with depth, we see a decrease of energy level with depth across the arrays and even within individual arrays at the lower and mid continental slope, where the energy level of the temperature fluctuations also decreases with increasing water depth (Fig. 3). Clear tidal peaks at diurnal (ca. 1 cycle day^-1^, marked K1) and semidiurnal tidal frequencies (ca. 2 cycles day^-1^, marked M2) are visible at the two shallower arrays, the oceanic plate and the mid continental slope array (Fig. 3A,C). Only the shallowest station of the lower continental slope array at ~ 5030 m water depth exhibits a tidal peak that appears at semi-diurnal frequencies (Fig. 3A), suggesting that below 5000 m depth the weak stratification in the bottom boundary layer hinders the detection of internal tidal waves in the near bottom temperature records. Similarly, a stretched peak at 0.3–0.4 cycles day^-1^ (marked P2) is only visible in the shallowest of the mid continental slope stations (Fig. 3C), implying that corresponding processes do not reach water depths larger than 2800 m (Fig. 1C). In contrast to these peaks that show a depth dependence across arrays, the internal frequency tide (marked P3) and the wide peak visible in the spectral estimates at frequencies of around 0.1 and 0.125 cycles day^-1^ (i.e. ~ 10 and ~ 8 days periodicity, marked P1) at the mid continental slope site weaken with increasing distance to the coast (compare Fig. 3D). Taken together, these observations suggest that the 0.1 and 0.125 cycles day^-1^ peaks of the spectral estimates are related to topographic Rossby waves that are trapped against the continental slope, which play an important role in the intraseasonal variability of isotherms along the Peruvian and Chilean continental margin^16,17^.

### Spatial fluctuations

A decrease in the amplitude range of the temperature oscillations with depth is observed both across arrays and within an array (Table 1, Fig. 2), as would be expected based on the decrease in the temperature gradient with depth. This relationship is most pronounced along the continental slope (Fig. 2B,C), due to the greater depth range within the slope arrays (~ 350 m and ~ 250 m) compared to the array on the oceanic plate (~ 75 m) (Fig. 2A,B).

**Figure 4:**
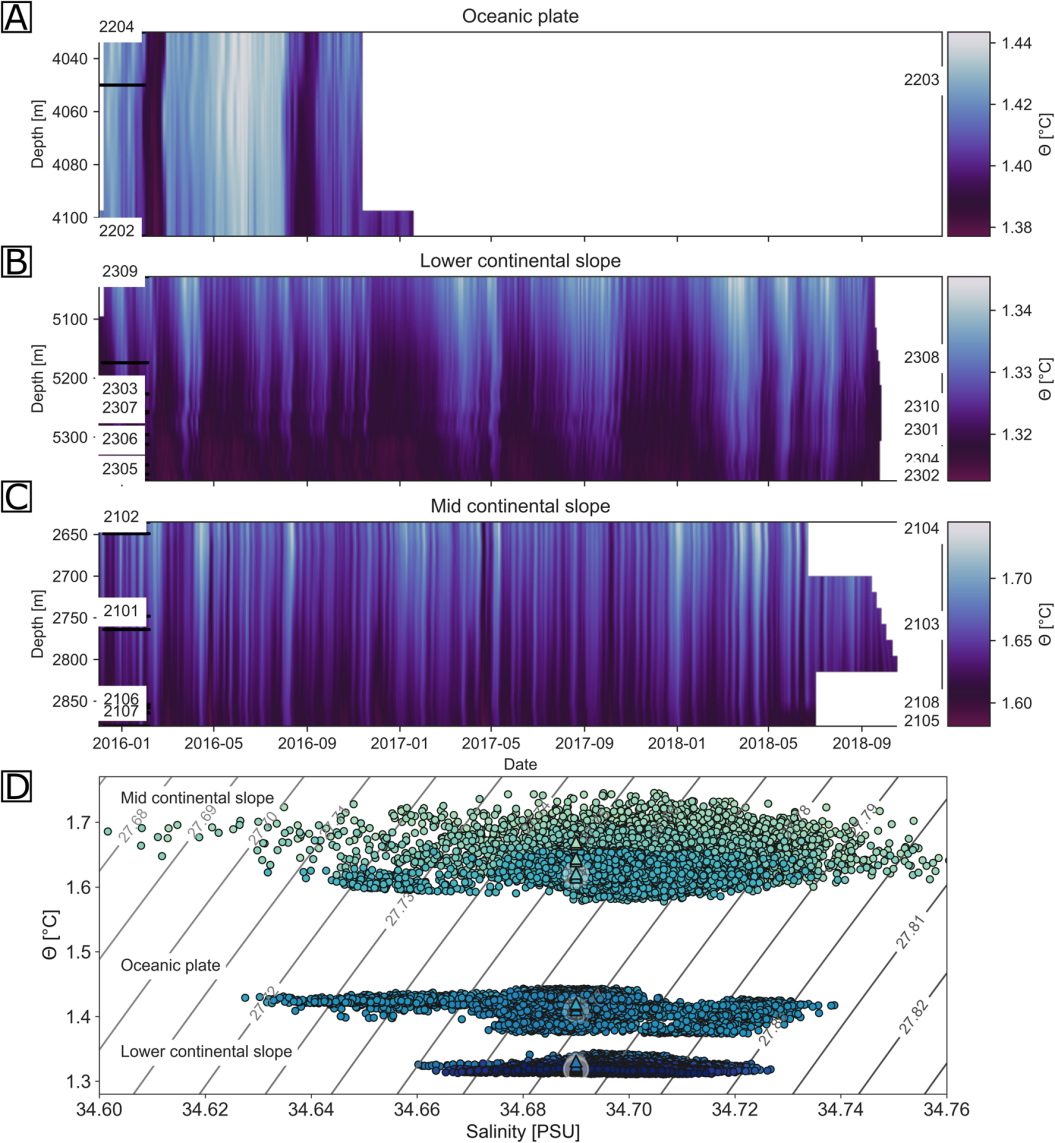
Temperature salinity plots. T/depth section of the three seafloor array sites (Panels (**A**–**C**)). The potential temperature was gridded with a grid spacing of one day along the horizontal axis and a grid spacing of 20 m along the vertical axis. The vertical axis displays the absolute depth, which is derived from AUV bathymetry and the station locations that are known from ROV dives. (Panel **D)** shows the T/S diagrams of all three arrays. Data points of each station are colour-coded based on the station’s relative depth, ranging from pale green at the mid continental slope to dark blue at the lower continental slope. The regional mean temperature-salinity from GLODAPv2 is indicated by a triangle also colour-coded in accordance to the station’s relative depth. Grey contour lines of potential density anomaly are added.

The periodic temperature fluctuations are visible as warm or cool anomalies in the temperature/depth sections (Fig. 4A–C) that were constructed for each of the arrays. While the events stretch through the sensor field, the event’s amplitude varies with depth as is particularly evident at the shallow mid continental slope (Fig. 4C), where the anomalies weaken with depth and show an absolute amplitude decay of <  = 0.15 °C, over 245 m. The visible coherence of the lower continental slope’s and the mid continental slope’s periodic temperature fluctuations cannot be found at the oceanic plate site, where partly shorter time series (due to sensor problems) and a comparably small depth range of the array lead to less variability between sensors (Figs. 3A,B, 4A,B).

The mid and lower continental slope temperature records show a more pronounced intraseasonal variability (fluctuations with periods of a about a week to a few months [or from ~ 7 to 90 days]), while the oceanic plate site records show much lower frequency variability. Making use of the sound speed observations, the salinity is estimated^18^ (see Supplementary Material, Fig. S6), which in turn enabled calculating the seawater density. The temperature/salinity diagram (Fig. 4D) reveals that the observed temperature fluctuations are not isopycnal and that the range of density fluctuations increases with shallower water depth, in line with the increase in vertical gradients towards the surface.

**Figure 5:**
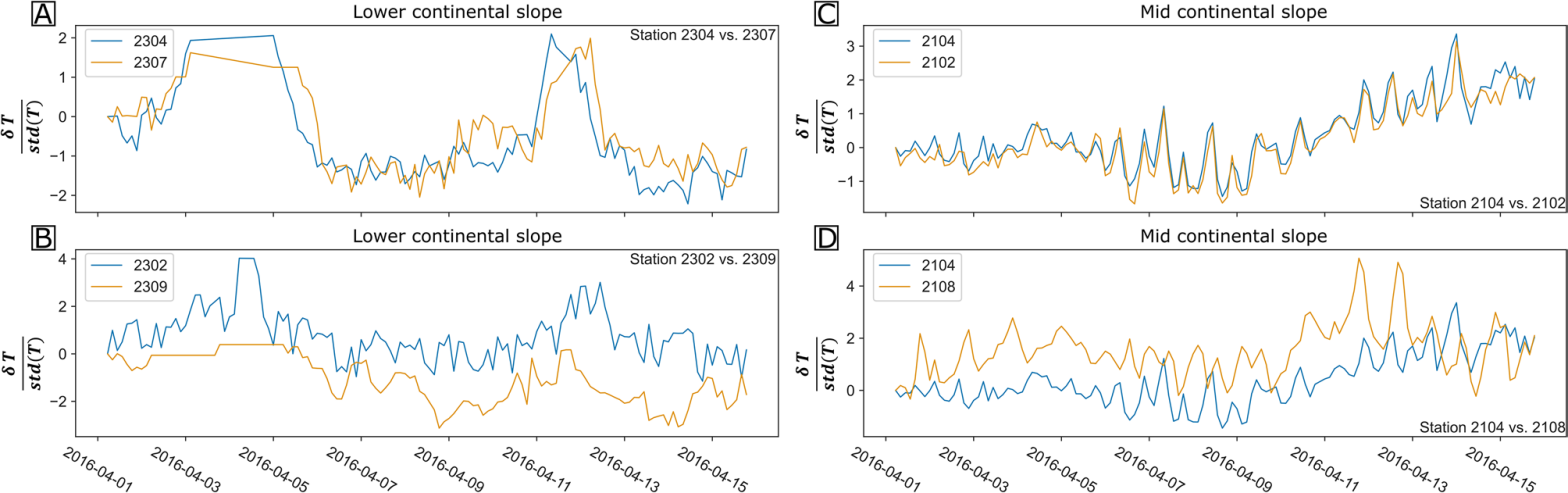
Exemplary tracking of normalised temperature anomaly event. Comparison of potential temperature changes between 01 April to 15th April 2016. Potential temperature is shown after subtraction of the first measurement of the selected time window and division with the standard deviation of the full time series. Panels (**A**,**B**) show temperature changes measured at stations from the lower continental slope array, while panels (**C**,**D**) compare temperature changes measured at stations from the mid continental slope array. Stations with maximum latitudinal and minimal longitudinal offset were chosen for panels (**A**–**C**), while stations with minimal latitudinal and maximal longitudinal offset chosen for panel (**B**–**D**).

Subsequently, the timing of warm anomalies was tracked through the individual arrays. Events associated with warm anomalies were used because they often displayed a more distinctive onset. The onset and amplitude modulation of individual anomalies was compared across arrays, which allowed us to derive information about the propagation of anomalies through the arrays and eventually mesoscale variability (Fig. 5).

The comparison of anomalies that were recorded at stations with roughly the same longitude (Fig. 5A,C) reveals that the shape of anomalies shows little latitudinal variation within an array. There is, however, a visible delay in the onset of the anomalies in between stations from the lower continental slope (Fig. 5A) as well as at the mid continental slope (Fig. 5C), though less pronounced. The delay in arrival at that the southern stations, compared to the northern, suggest that anomalies propagate from north to south over the continental slope. At the continental slope sites, the shape of the anomalies appears increasingly stretched with growing distance to the coast and water depth (Fig. 5B and D). While this stretching of anomalies is apparent even on an intra-array scale at the lower continental slope site (Fig. 5A,B), no obvious changes in the shape of the anomalies are visible in between stations of the mid continental slope data (Fig. 5E). We relate the absence of significant spatial dependencies to the overall smaller depth range the mid continental slope array (~ 200 m) compared to that of the lower continental slope array (~ 400 m). Similarly, the limited extent of the oceanic plate array is likely to inhibit the detection of spatial dependencies of the anomaly shape and offset at the oceanic plate site. However, the substantial differences in the shape of the warm anomalies between the arrays (Fig. 5), indicate a decoupling between the dynamical regimes on the oceanic plate and the continental slope.

### Interannual warming trends

Apart from the seasonal component, there is a linear warming trend observed in the temperature data from the lower continental slope (Fig. 2B). No visually discernible linear trend could be observed in the temperature series from the oceanic plate site or the mid continental slope (Fig. 2A,C), likely related to the larger amplitudes of the seasonal signal component, which would obscure a comparatively weak linear trend in a 2.5-year long time series. Therefore, linear regressions were calculated for the temperature data of all three arrays (Fig. 2, dashed lines). Negative trends were determined for three of the five oceanic plate stations, with trends ranging between − 0.0058 °C yr^−1^ and 0.034 °C yr^−1^ and an average trend of 0.010 °C yr^−1^. In contrast, the majority of the linear regressions performed on the temperature series from the lower continental slope, suggest a positive trend, with determined gradients ranging between 0.000 °C yr^−1^ and 0.002 °C yr^−1^ with an average trend of 0.002 °C yr^−1^. Comparable high-resolution temperature trends that range between 0.001 °C yr^−1^ and 0.006 °C yr^−1^ (average of 0.003 °C yr^−1^) were determined for the mid continental slope temperature series. The strong amplitudes of the periodic temperature fluctuations on the oceanic plate and the mid continental slope, as well as the shorter record lengths on the oceanic plate, however, lead to larger uncertainties in determining the interannual temperature trend (Fig. 2).

**Figure 6:**
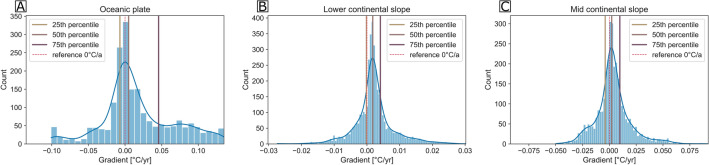
Histograms of interannual warming trends. A total of 400 randomly chosen subsets were extracted from each station’s high-resolution temperature series and had linear regressions fitted to them. The 400 trends thus determined per station were grouped by array and are shown in panels (**A**–**C**) for the oceanic plate and the lower and the mid continental slope respectively. The kernel density estimation (blue line), a 0 °C/a reference line (dashed red line) and the 25th, 50th and 75th percentile are superimposed onto the histograms.

In order to test the independence of the positive linear trend from the duration of the temperature series, subsets of the temperature series were selected and used for the calculation of linear regressions. Figure 6 shows the histograms of the temperature trends, determined by the linear regressions of the high-resolution temperature series subsets of all three arrays. On the oceanic plate, the determined trends are distributed multimodal and show substantial mean variations around the average trend of 0.018 °C yr^−1^ (Fig. 6A). The determined trends range from − 0.101 °C yr^−1^ to 0.190 °C yr^−1^. The side peaks in the high-resolution temperature histogram are possibly the result of seasonal effects that are likely to dominate the subsets of the two short high-resolution temperature records. At the lower continental slope, the calculated temperature trends range between − 0.026 °C yr^−1^ and 0.028 °C yr^−1^ with an average trend of 0.002 °C yr^-1^ (Fig. 6B). A comparable mean temperature trend of 0.003 °C yr^−1^ was determined at the mid continental slope, though trends showed a larger variance with trends ranging between − 0.050 °C yr^−1^ and 0.091 °C yr^−1^ (Fig. 6C). Even though the determined interannual temperature trends are very small, we are confident that the determined temperature trends represent physical changes in ocean properties and not sensor drifts (see Supplementary Material).

## Discussion

High-resolution temperature series of about 2.5 years were acquired at 23 deep sea seafloor stations that were clustered in three arrays spaced along a roughly E-W trending transect crossing the Chilean trench system (Fig. 1D). All stations were equipped with a high-resolution temperature sensor, a pressure sensor as well as a sound speed sensor and were deployed with average station distances of less than 1 km (Fig. 1). One sensor array was deployed on the oceanic plate and thus seaward of the deep-sea trench. Two sensor arrays were placed on the lower and mid continental slope (Fig. 1C). All arrays are located in the deep ocean with water depths ranging between 2600 and 5500 m. The stations deployed on the lower continental slope have a horizontal distance to those deployed on the oceanic plate by about 76 km and to those deployed on the mid continental slope by 26 km. The data, originally acquired for geodetic analyses, are the first multiyear direct observations of bottom layer temperature variability in the eastern Pacific and were used successfully to assess the spatial and temporal variability in the deep ocean offshore northern Chile.

Recent studies using ocean models and direct current observations have found that strong and seasonal currents also occur in the deep ocean^16,19–23^. These studies suit the periodic temperature fluctuations that have been observed in this study and whose southwards increasing onset times are understood as indicative for an overall southwards flow of bottom water over the lower and mid continental slope off northern Chile. By deriving salinity from the sound speed observations and in turn calculating the potential density, it could be shown that the temperature fluctuations are associated with a wide density range (Fig. 4). The periodic fluctuations align with what is expected from tidal and topographic wave-induced displacements that are evident in the spectral analysis of the time series (Fig. 3). The presence of such spatial and temporal temperature heterogeneities is particularly severe for high-resolution or long-distance soundwave-based localisation systems (as used in seafloor geodesy studies), which usually assume a constant speed of sound^24^. While the speed of sound in water is controlled by the ambient temperature, pressure and salinity, it is most sensitive to temperature changes^25^.

Fluctuations of surface currents off the Pacific South American coast have been studied extensively with satellite altimetry, hydrographic sampling and sea surface temperature data^26–29^. Together with alongshore subsurface current observations on the shelf and continental slope off northern and central Chile, these studies determined strong, low-frequency currents—and sea level fluctuations, which have been associated with coastal-trapped waves that propagate southwards along the Chilean coast^16,27,30,31^. A 10-day fluctuation, similar to that observed in all spectral estimates from the mid—and lower continental slope (Fig. 3B,C), has been found in other boundary current systems and has been linked to coastal-trapped waves that are forced mainly by eastward propagating, baroclinic, mixed Rossby-gravity waves that are trapped around the equator^32,33^. Offshore Peru, these coastal-trapped waves could be traced as far as 15°S, from where on, data sparsity southwards to 30°S has impeded the tracking of the waves to date. Because no phase velocities could be determined from the available data, we cannot definitively resolve whether the 0.1 cycles day^−1^ temperature fluctuations are related to remotely forced coastal-trapped waves or are linked to coastal-trapped waves forced by the wind along the South American coast. In agreement with coastal-trapped wave theory, the energy of the ~ 0.1 cycles day^-1^ temperature fluctuations decrease visibly over the 26 km separating the mid and lower continental slope arrays and is negligible on the oceanic plate (Fig. 3). This absence of a 10-day periodicity at the oceanic plate site seaward of the deep sea trench (approx. 167 km from the coastline) would also coincide with observations made along the Chilean coast at 30°S, where current meter data conveyed that these large period fluctuations are strongly bound to the coast and could not be traced as far seaward as 150 km offshore^31,34^ or to the oceanic plate domain.

Previous studies that targeted the deep and abyssal ocean with repeated measurements of full-water-column-hydrographic sections have described considerable warming of abyssal waters^35–37^, which appears most prominent in the Southern Ocean and weakens northwards^38^. The rate of warming has been suggested to accelerate over time, based on observations from the southwest Pacific of 0.001 °C yr^−1^ in 1990–2000 to 0.003 °C yr^−1^ in 2014–2018^39,40^. Deep-sea temperature changes of the Pacific Ocean are typically attributed to either advection of warmer waters directly from the source region in the Ross Sea, or changes in source water formation regions (e.g. ^26,27,32^). Since the interannual temperature trends that were determined over the continental slope (0.002 °C yr^−1^–0.003 °C yr^−1^) are of the same magnitude as published deep-sea warming trends, the observed bottom water warming is likely related to similar processes. The bottom water of the Chile basin is dominated by Lower-Circumpolar Deep Water masses that enter over the Chile Rise from the west and through morphological gaps in the Chile Rise from the south, from where they are known to circulate cyclonically flowing southward on the eastern side of the basin^41–43^. Because no deep circulation pattern of higher resolution than the basin-scale cyclonic gyre^41,42^ has been resolved for the study area and we are not aware of any deep current meter data, a more detailed pattern of deep currents cannot be resolved. However, the concurrent change in the shape of the warm anomalies and the rate of deep-sea warming that occurs with the crossing of the deep-sea trench (Figs. 5 and 6) suggests that the abyssal part of the eastern boundary current system off Chile does not extend past the deep sea trench. Therefore, even though differences in the interannual temperature trends could be partly related to increased uncertainties, a deviation in ventilation time scales and surface ocean connectivity would imply a decoupling of dynamical regimes over the deep sea trench, which could potentially have implications for other deep sea trench systems.

While surface currents may be studied using remote sensing techniques, our knowledge and understanding of deep sea oceanographic regimes must depend on in situ observations and measurements. To this end, our original motivation was to study the feasibility and benefits of coupled deep ocean oceanography-geodesy experiments. Deep ocean observables measured in geodetic experiments provide input parameters for hydrographic studies, prompting a debate on coupled experiments between oceanography and offshore geodesy. The benefit of such coupled experiments would be cross-beneficial. The benefit for the geodetic community lies in the potential the extensive oceanographic calibration of offshore geodetic data has to increase the accuracy with which sound speeds can be determined and, therefore, the practical resolution of geodetic experiments^44^, while the benefit for the oceanographic community lies in the gratuitous, high-resolution monitoring of oceanographic parameters.

In summary, the data of a geodetic experiment of the deep ocean offshore northern Chile, were analysed for deep ocean hydrographic variability, revealing previously unknown de-coupled dynamical regimes seaward and landward of the deep-sea trench, periodic temperature anomalies as well as interannual warming trends. However, the data did not just allow a full hydrographic study, but enabled us to uncover heterogeneities across distances, where bottom water properties are traditionally considered to be uniform and thus demonstrates the potential of cross-disciplinary experiments between offshore geodesy and oceanography.

## Materials and methods

Seafloor geodetic sensor arrays were deployed across the Chilean deep-sea trench system in late November to early December 2015. The sensor arrays consisted of 23 seafloor stations (Fig. 1) and were visited twice before their recovery in 2022, in order to upload data using an acoustic modem, once between October and December 2016 and a second time in June 2018. Each sensor array had between five and ten seafloor stations, which were each equipped with an acoustic ranging device, an inclinometer, a pressure sensor and a high-resolution temperature sensor (see supplementary material for more detail). The instrument was mounted on top of a 4 m high tripod. The first array was deployed on the oceanic crust of the Nazca plate and consisted of 5 stations located at distances between 185 and 823 m, covering about 0.352 km^2^ (Fig. 1A). The oceanic plate array is located on the oceanward side of the Chilean deep sea trench, about 167 km away from the coast, at water depths between 4.0 km to 4.1 km. The second array, which consisted of 10 stations, was deployed on the lower continental slope, i.e. on the landward side of the deep sea trench and approximately 92 km away from the coast (Fig. 1B). The stations of the lower continental slope array were spaced 491 m–3961 m apart and covered a surface of roughly 7.137 km^2^, at a water depth between 5.1 km and 5.4 km. The third and last sensor array was also deployed on the continental slope at about 67 km distance to the coast and is referred to as the mid continental slope array (Fig. 1C). The mid continental slope array consisted of 8 stations, which were spread over an area of 2 km^2^, with interstation distances ranging between 63 and 2729 m.

All of the deployed stations acquired high-resolution temperature, pressure and sound speed data. A measurement was executed every 90 min at the oceanic plate site, and every 160 min at the lower and mid continental slope. Hence, in order to ensure comparability, data from the oceanic plate site were resampled with a 160 min sampling interval. Acquired temperature records include one record that was measured with a high-resolution temperature sensor and another, independent temperature record that was acquired by the pressure-sensor-internal temperature sensor. Since the pressure-sensor-internal temperature sensor is much less accurate than the high-resolution temperature sensor, the authors focus on the high-resolution temperature data in this study. However, information regarding the pressure-sensor-internal temperature sensor data can be found in the supplementary material. The acquisition of temperature data was started shortly before the deployment of the stations, which took place between the 30. Nov—05. Dec. Station deployment started on the oceanic plate, after which the lower continental slope stations and the mid continental slope stations were deployed. In order to exclude data that was acquired before deployment or acclimatisation of the stations, the first few days of data acquisition were not considered in further analyses. On the oceanic plate, only records from after 02. Dec. 2015 were considered, while only records from after 07. Dec. 2015 were considered at the lower continental slope and records from after 08. Dec 2015 were considered at the mid continental slope. The length of the acquired temperature series varies most on the oceanic plate, where two temperature sensors acquired just under one year of temperature data (2201, 2202), while two other stations acquired just under two years of temperature data (2203, 2204) and one station (2205) stopped data acquisition after about a month. At the lower continental slope, most of the sensors recorded until late July or early November 2018 and therefore acquired just under three years of data. Only three lower continental slope temperature sensors (stations 2301, 2305 and 2310) stopped recording prematurely in late 2017 to early 2018. At the mid continental slope, all temperature sensors acquired data for 2.5–3 years and thus recorded until mid to late 2018 (May-December). Further discussion of the instrumentation, as well as the estimated sensor resolution and CTD data can be found in the supplementary material.

The measured in-situ temperature was converted to potential temperature using the conversion tools from the Gibbs SeaWater Oceanographic Package of TEOS-10 conversion tools, using a a reference pressure and a constant salinity salinity equivalent to PSS-78 34.69 PSU. This reference salinity was assumed for all stations, as the value represents the rounded salinity value measured at the deepest point of all the arrays’ CTD profiles (see supplementary material, Fig. S5). In order to improve the accuracy of the measured temperature, pressure and sound speed data, we applied a calibration of the individual time series. For the calibration, the average value of the corresponding time series is shifted to fit the reference value for each station of the three arrays. By doing this, the accuracy of the time series is increased without losing the time series’ information on spatial or temporal variability. For pressure data, the reference value is predicted by estimating the pressure at the station’s depth, known from AUV bathymetry and using a latitude dependent conversion function of the seawater python libraries. For temperature, the reference value was predicted through a second order polynomial, which was fitted to potential temperature and pressure data of the study area extracted from GLODAPv2. The thus determined polynomial was then used to predict the potential temperature at the corresponding station, to which the measured time series’ average was then shifted for bias correction. For sound speed, we determined a reference sound speed using a conversion function of the seawater python library that made use of the calibrated temperature and pressure time series and a constant PDD-78 salinity of 34.69 PSU. However, additional corrections had to be applied to the sound speed data after the average calibration, because sound speed sensors are prone to outliers, caused typically by the ageing of sensor components, biofilm development around the sensor, or (bio)matter suspended in the water column. For the outlier-correction, third order polynomials were estimated, which described the relationship between the calibrated potential temperature and the detrended and average calibrated sound speed measurements of each station. Sound speed measurements were set to the value predicted by the polynomial, if the measurement was offset from the predicted sound speed by more than 3 standard deviations.

Salinities were derived from the calibrated sound speed data using a grid search algorithm, in which the misfit between measured and predicted sound speed data was minimized. At every time step, a range of salinities (0–40 PSU) were used together with the measured temperature data and a constant reference pressure, derived from the station’s depth, to predict a sound speed, which was then compared to the measured sound speed. If a predicted sound speed value was offset to the measured sound speed by less than 0.0001 m/s, its associated salinity was assumed to be representative of the water properties at the corresponding station and time. Subsequently, derived salinities that were offset to the rolling average (window size: 4.5 months, minimum period = 7 days) by more than 1 standard deviation were dropped from the salinity time series.

The seasonal component of the data was analysed by calculating the spectral estimate using the multitaper method using prolate spheroidal (Slepian) tapers (Fig. 3), as implemented in the spectrum python library^45^. Multitaper spectral analysis was chosen, because it introduces comparatively little broadband bias to the spectral estimates. Broadband bias (also referred to as broadband leakage) is a noise source which is generally inevitable when estimating spectra of finite time series, but can be reduced by application of tapering windows^46–48^. Further benefits of the multitaper spectral analysis arise from the consideration of multiple taper windows, the orthogonal eigenspectra of which are averaged during the spectral analysis, effectively reducing the variance of the spectral estimate^46–48^. In the performed multitaper spectral analysis, the first 19 Slepian sequences were used with a time half bandwith parameter of 10, as it appeared as optimal combination considering the bias-variance trade-off. Prior to the application of the multitaper method, all temperature time series were shortened to the same length (225.3 days) so that the minimum resolvable frequency would be the same in all spectral estimates. Therefore, since all the time series had already been resampled to with the same sampling interval (160 min), the resolvable frequencies of all power spectral estimates lie between 0.004 cycles day^−1^ and 5 cycles day^−1^. Shortening every time series to the same length had the additional benefit that spectral blurring related to the applied half-band width of the tapering window is the same for all the considered time series.

Because the stations of each sensor array were spread out over a range of water depths, the vertical stratification of the water column can be studied with the data, but only if it is assumed that the horizontal gradient of the stratification can be neglected over the hundreds of metres of offset in between the stations. For the analysis of the vertical stratification, the potential temperature data of all stations of an array are sorted by depth and gridded, with a grid size of one day in the x direction and a grid size of 20 m in depth. This temperature matrix was converted to potential temperature (Fig. 4A–C). Likewise, salinities were derived from the calibrated sound speed data. A T-S diagram was plotted using the derived salinities and potential temperatures (Fig. 4D), however because of its record length of only about a month, station 2205 was excluded from the analyses. To ensure comparability, all station’s time series were shortened to that of the station with the shortest, considered record length (2201). Hence, the data of approximately 7 months is shown in the T-S diagram (Fig. 4D). The propagation direction was determined by comparison of the onset of multiple warm anomalies between stations of an array. While one example is shown in Fig. 5, more can be found in the supplementary material (Fig. S1).

In order to estimate an interannual temperature trend for each station, linear regressions of the entire time series were performed. Before the linear regressions were calculated, a moving average with a window size of 7 days and a minimum period of three days was applied to the data in order to reduce the effect of noise and daily variations. After linear regression of the entire time series, a more stochastic approach, in which the time series were split into random subsets before calculation of the linear regression, was performed. The stochastic approach was undertaken in order to ensure the independence of the determined interannual temperature trend from the record length and timing. A total of 400 random subsets were extracted from each station’s temperature series and linear regressions were fitted to each. The distributions of the thus determined temperature tends are illustrated array-wise in Fig. 6, though interpretations focus on the average trend thereafter. Each of the extracted subsets was chosen randomly, meaning that the subsets could overlap and vary in length. The only restriction that was imposed in the random subset selection was that subsets had to have a minimum length of about half a year.

### Supplementary Information

Supplementary Information.

## Data Availability

The multibeam bathymetry grid (https://doi.org/10.1594/PANGAEA.893033), as well as the pressure, temperature and sound speed data of all 23 stations (https://doi.pangaea.de/10.1594/PANGAEA.961701) are accessible on PANGAEA.
