# Supplementary material for: Deep ocean hydrographic variability estimated from distributed geodetic sensor arrays off northern Chile
Source: Sci Rep. 2024 May 15;14:11163. doi: 10.1038/s41598-024-61929-z (PMC11096396; doi:10.1038/s41598-024-61929-z)
Supplement: Supplementary file 1 — Supplementary Information. [file 41598_2024_61929_MOESM1_ESM.docx]

Supplementary Materials for

Deep ocean hydrographic variability estimated from distributed geodetic sensor arrays off northern Chile

Authors

Anna Jegen^1^*, Dietrich Lange^1^, Johannes Karstensen^1^, Oscar Pizarro^2^, Heidrun Kopp^1^

*Corresponding author. Email: [ajegen@geomar.de](mailto:ajegen@geomar.de)

Affiliations

1GEOMAR Helmholtz-Zentrum für Ozeanforschung Kiel, Kiel, Germany.

2Universidad de Concepción, Barrio Universitario s/n, Concepción, Chile.

**This PDF file includes:**

Supplementary Text

Figs. S1 to S6

References

Supplementary Text

Instrumentation details of the high-resolution temperature sensors

A custom Pt100 design, developed by Sensing Devices Ltd is housed in the pressure tubes of the geodetic sensors. Each of the 23 instruments was mounted onto a high steel frame so that all data was acquired 4 m above the seafloor. The resolution has been determined to conform to 1/10 of the DIN Class B, translating to a tolerance of +/-0.025 °C. Low drift components were used in all signal conditioning electronics, such as the AD converter, the voltage reference in the AD converter and the calibration resistor. In temperature sensors such as the Pt100, the most common cause for sensor drift is related to the ageing of the reference voltage used by the A/D converter, which is also known as long-term drift (LTD). The LTD curve of a typical device is attenuated with time. The precise time range that has to pass before the LTD settles within a low drift coefficient is temperature dependent and increases with decreasing temperature. The exact approximation of the LTD’s effect on the data is hindered by the nonlinear behaviour of the reference voltage that is not consistent between devices nor between different kinds of reference voltages. Nevertheless, since the same build of reference voltage was used in all stations of all three arrays, the general course of the LTD should be similar, since they were deployed at roughly the same time and in water of comparable temperature. Therefore, since the discussed linear warming trend is only detectable in two of the three arrays, and all stations are of the same build and age, we conclude that the trend is not the result of the LTD, but records a physical change in water properties. Other experiments, which used stations of the same build in the Marmara Sea and Ionian Sea showed that while temperature increases and concurrent travel time decreases were measured in the Marmara Sea, temperatures in the Ionian Sea appeared to stagnate ^1–3^. The analysis of the relative course of the temperature records from one array, has shown that the intra-array variability is not random, but systematic, which suggests that the variability is not the result of lacking sensor accuracy but linked to the depth-dependent decrease of temperature field fluctuations (Fig. 1B). Since the smallest intra-array variability that could be observed is much smaller than the official tolerance of +/- 0.025 °C (e.g. ~0.0017 °C, Fig. 1B), we conclude that the accuracy of the temperature sensors must be smaller or equal to the observed intra-array variability.

Pressure-sensor-internal temperature sensor data

Every station was equipped with a pressure sensor, which acquired absolute pressure data at the same rate and time as the temperature was measured (160 min at the mid and lower slope; 90 min at the outer rise). The pressure sensors that were mounted to the stations were manufactured by Presens and have an internal Pt1000 temperature sensor. The internal temperature sensor is needed for the conversion of the pressure sensor’s internal engineering units to physically meaningful units ^4^. However, since this conversion merely requires a temperature approximation, pressure-sensor-internal temperature sensors are seldom calibrated absolutely and are thus of lower accuracy than stand-alone temperature sensors. Nevertheless, potential temperatures were calculated from the pressure-sensor-internal temperature measurements using Gibbs SeaWater Oceanographic Package of TEOS-10 and reference values for salinity and pressure. The increased degree of chaotic intra-array variability that is particularly visible at the lower slope (Fig. S1B), where temperature fluctuations are the weakest, is likely related to the decreased precision of the pressure-sensor-internal temperature sensors. In spite of the reduced precision of the pressure-sensor-internal temperature sensors, the corresponding records were still used for linear regression as they are an independent temperature observation and can thus aid in the verification of the observed temperature trends (Fig. S3).

The histogram of the pressure-sensor-internal temperature trends from the outer rise shows stronger side peaks and a larger variance than the histogram of the high-resolution temperature trends from the outer rise (Fig. 6A, Fig. S5A). At the two slope arrays, the stochastic linear regression of the pressure-sensor-internal temperature records determines average temperature trends that are larger than those determined by the stochastic linear regression of the high-resolution temperature records. However, with an average trend of 0.003 °C/a at the lower slope and 0.004 °C/a at the mid slope, the pressure-sensor-internal temperature trends are nevertheless of the same magnitude as the high-resolution temperature trends.

At the outer rise, the trends of the pressure-sensor-internal potential temperatures range between -0.003 °C/a and -0.001 °C/a with an average of -0.002 °C/a. At the lower slope, multi-year trends ranging between- 0.002 °C/a and 0.033 °C/a with an average of 0.003 °C/a were determined. Similarly, warming trends ranging between -0.051 °C/a and 0,092 °C/a with an average of 0,004 °C/a were determined for the pressure-sensor-internal temperature records from the mid slope. While the average warming trends, determined by the linear regression of the pressure-sensor-internal temperature records and the high-resolution temperature records are very similar at the mid (0.004 °C/a and 0.003 °C/a) and lower slope (0.003 °C/a and 0.002 °C/a respectively), average slopes from the outer rise (-0.002 °C/a and 0.018°C/a respectively) deviate stronger between sensors.


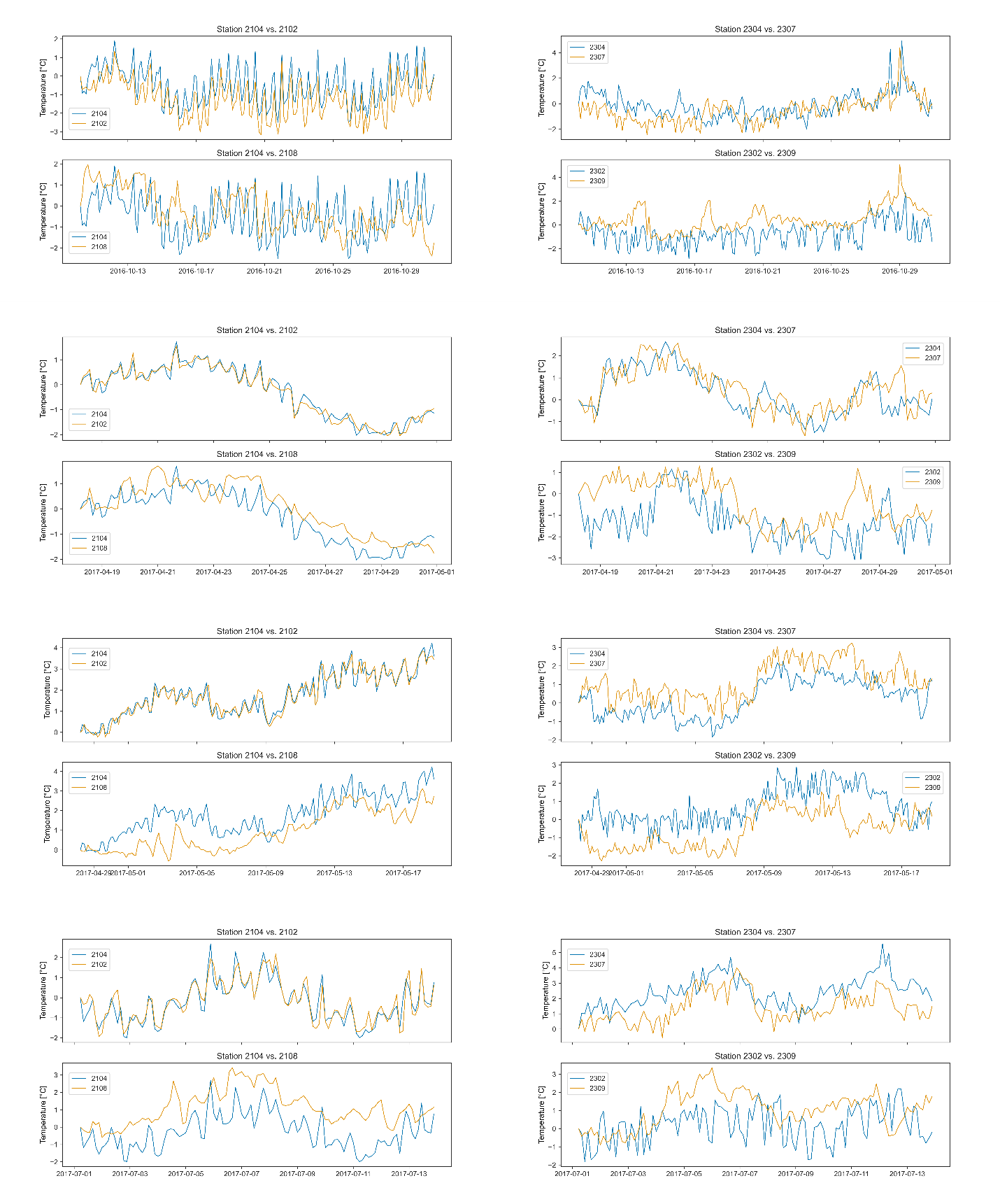


Figure S1.

Comparison of potential temperature changes during warming events of exemplary periods. Left column shows temperature changes measured at stations from the mid slope array and right column those measured at stations of lower slope array. Temperature changes are shown relative to the event’s first measurement and normalised with its standard deviation.


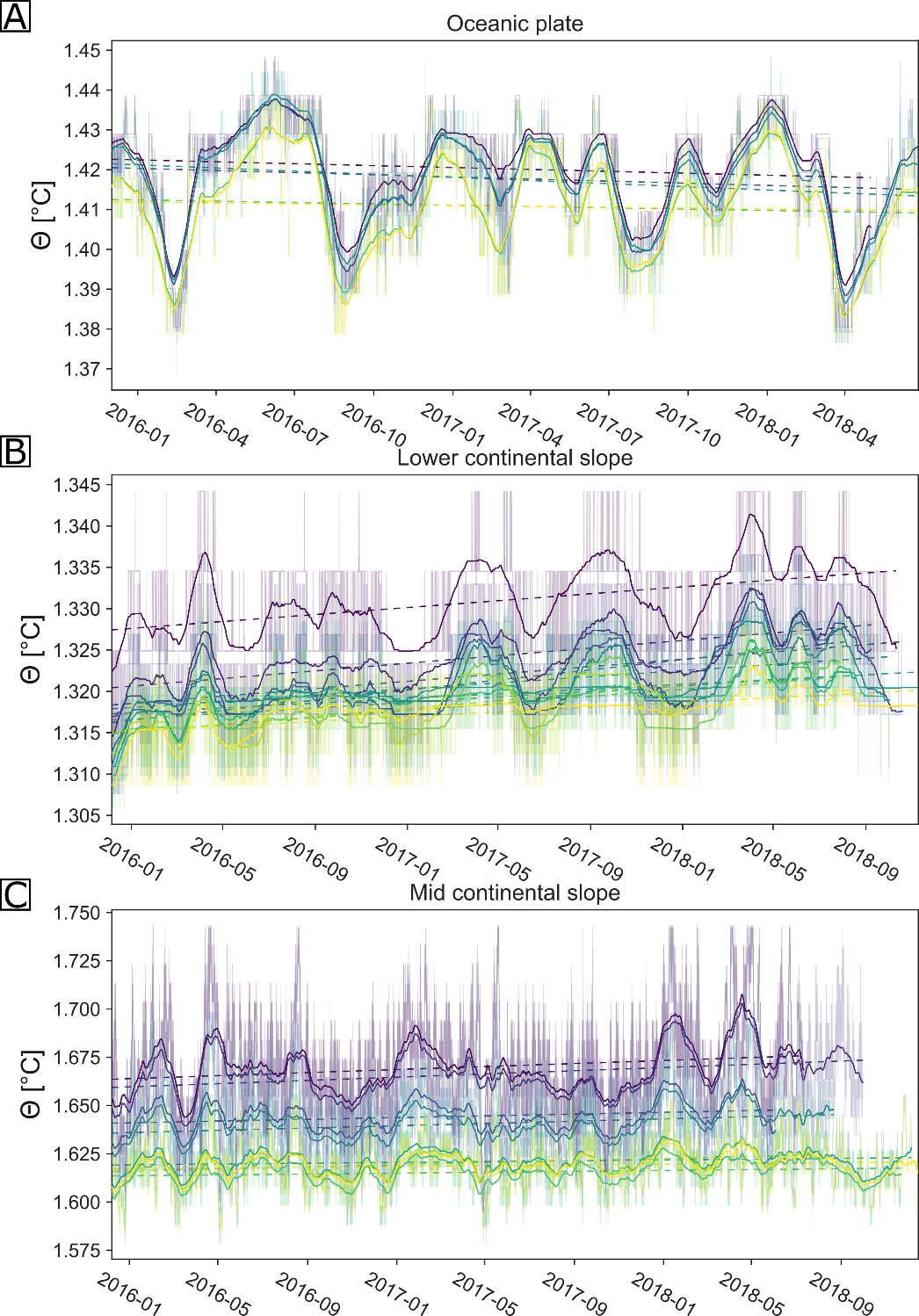


Fig. S2. Monthly running averages, with a minimum period of 7 days, were calculated from all station’s temperature records that were acquired by the pressure-sensor-internal temperature sensor. Panel A shows the outer rise array, panel B the lower slope array and panel C the mid slope array. The monthly average of each station is shown colour-coded according to the relative depth of the station. The multi-year temperature trend of each station, determined via linear regression of the entire time series is marked in the plot by a dotted line, coloured in accordance with the station’s measured potential temperature.


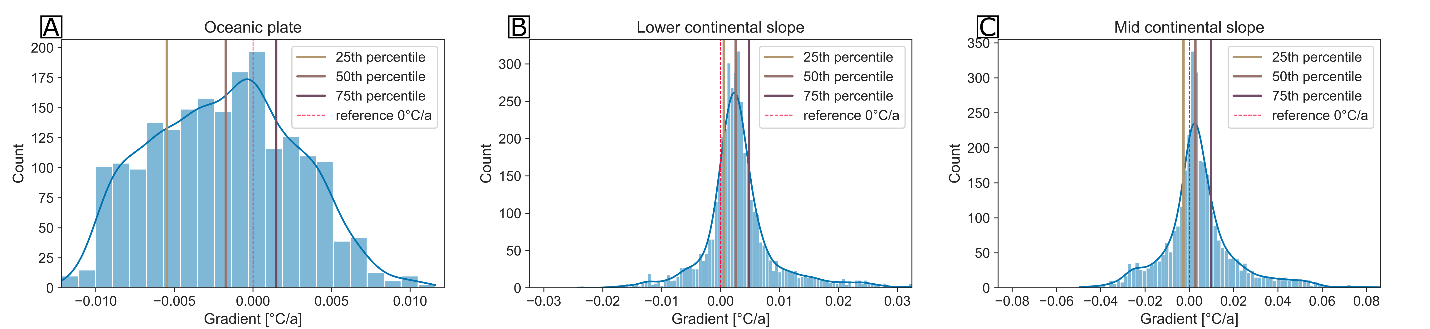
Fig. S3.

The histograms of the slopes (i.e. yearly temperature trends) calculated from the pressure-sensor-internal temperature series of the outer rise, the lower slope and the upper slope are shown in panel A, B and C, respectively. A total of 400 subsets were extracted from each station’s records and used for linear regression. The kernel density estimation (blue line), a 0 °C/a reference line (dashed red line) and 25th, 50th and 75th percentiles are drawn onto the histograms.


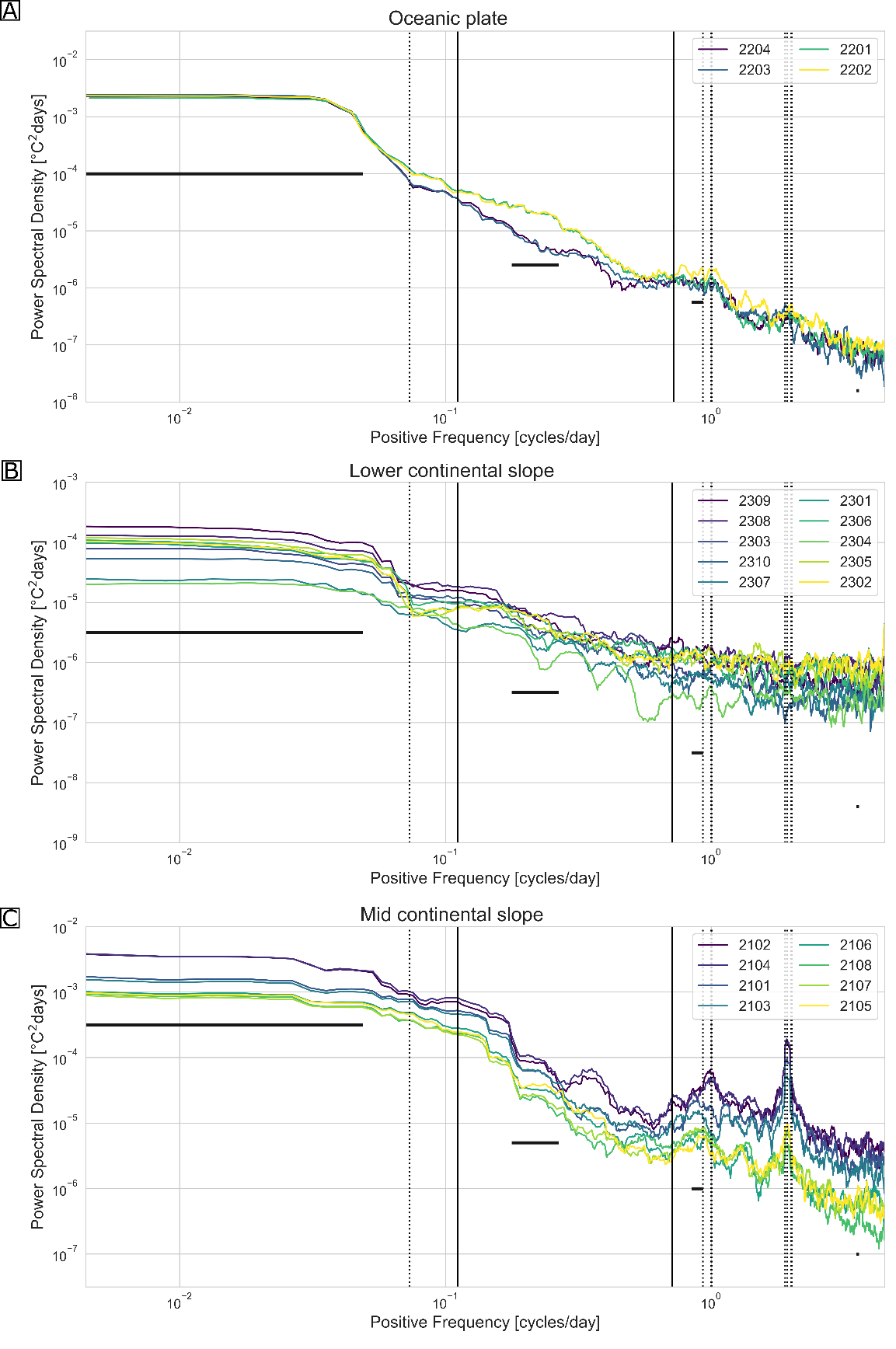


Figure S4.

Spectral estimate of all temperature series acquired by the pressure-sensor-internal temperature of each station of the three arrays (A-C). Spectra are estimated using the multitaper method and a time-bandwidth product of 10. The spectral estimates are coloured according to the station’s relative depth (shallow (green) to deep (blue)).


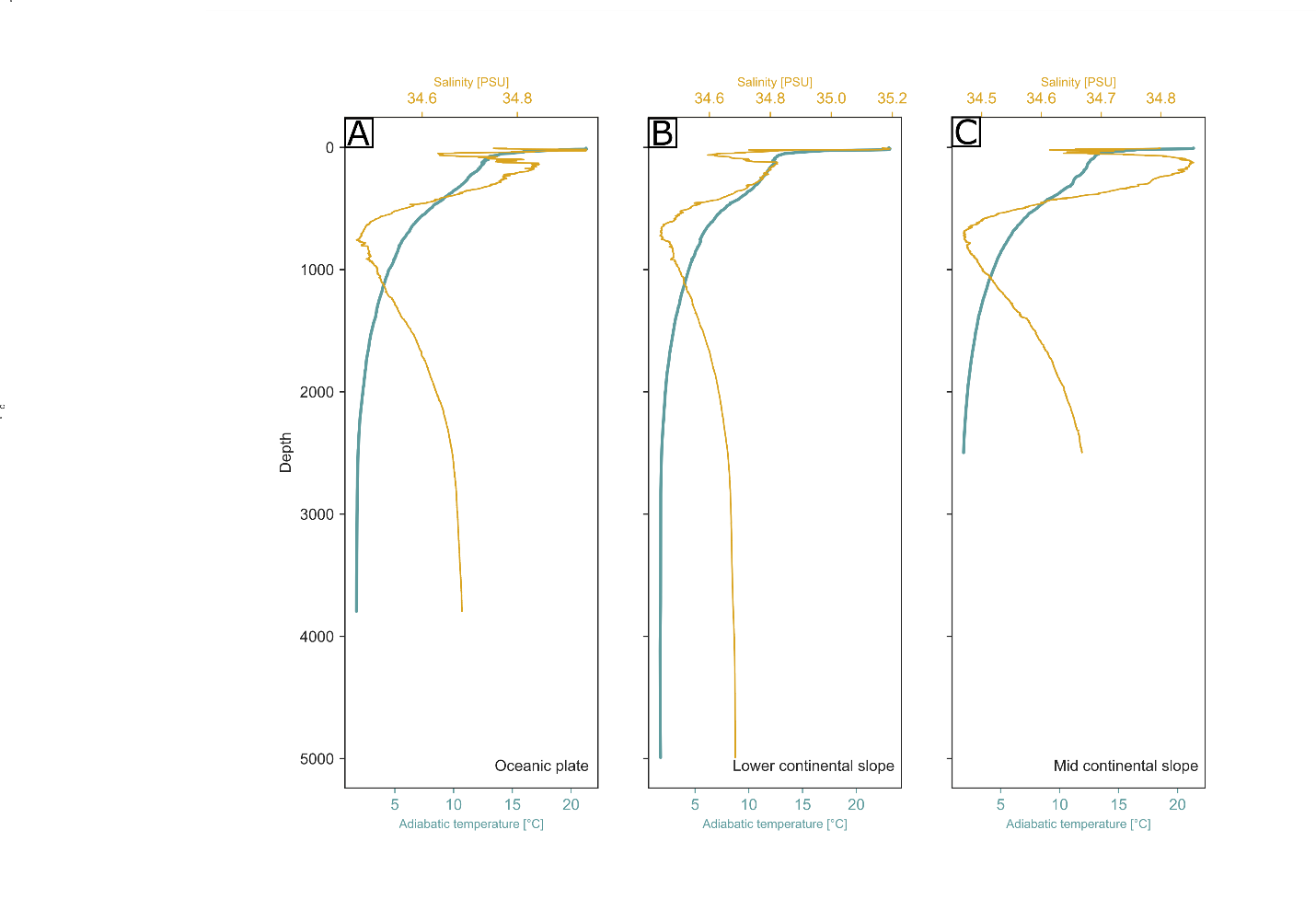


Figure S5.

Temperature and Salinities, measured by CTD casts acquired over the three arrays. CTD cast location from left to right is A: 21° 03.609’S, 071° 44.149’W; B: 20° 46.565’S, 071°04.631’W; C: 20° 47.887’ S, 070° 49.824' W.

Sound speed data and derived salinities

The discussed sound speed measurements were acquired by the built-in sound speed sensor of the acoustic transponder *Type 8300 Wideband Transponder* (manufactured by *Sonardyne*)*.* The built-in sound speed sensor is the 100 mm model, which is given a resolution of 0.01 m/s and an accuracy of +/- 0.03 m/s by the manufacturer.

The salinities derived from the sound speed measurements of each station are shown in Figure S6. The time series of an array show similar overall trends, though station 2310 and 2104 show oscillations that are not mirrored by the other stations and are thus possible artefacts. While the oceanic plate site appears to experience an overall salinity increase over the time of the experiment, salinities appear to decrease over the continental slope (Fig. S6). Over the continental slope, the most prominent features of the salinity depth sections are, the abrupt increase in salinity at the beginning of 2016 (Fig. S6B+C) and the anomalies produced by the salinity oscillations of 2310 and 2104 (Fig. S7+6B, 2017-05; Fig S7+6C, 2017-09). However, the corresponding salinity oscillations of stations 2310 and 2104 are likely artefacts, since they do not appear in the neighboring station’s salinities. High frequency oscillations, similar to those seen in the T/z section (Fig. 4), can be observed in the salinity data of all three arrays, though they appear to have larger average frequencies and lack the clear depth-dependency of the amplitude (Fig. S7).


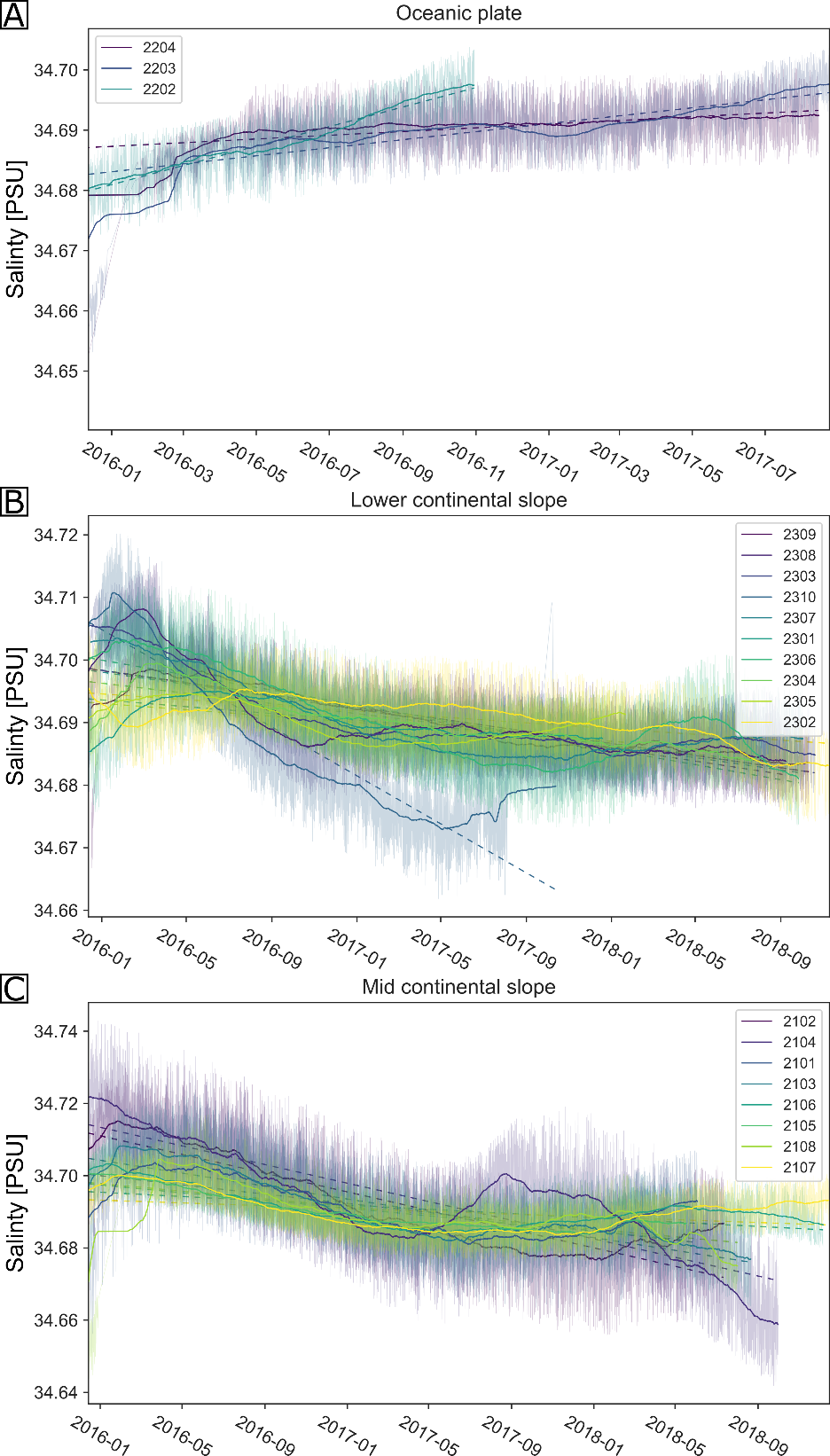


**Figure S6.**

Thin lines show the derived salinity data and solid lines the monthly running averages, that were calculated with a minimum period of 7 days. The monthly average of each station is shown colour-coded according to the relative depth of the station and the average of all the array’s temperature records was added in black. Panel A shows the oceanic plate site, panel B the lower continental slope array and panel C the mid continental slope array. The multi-year temperature trend of each station, determined via linear regression of each time series is marked by the dotted lines, coloured in accordance with the station’s derived salinity.


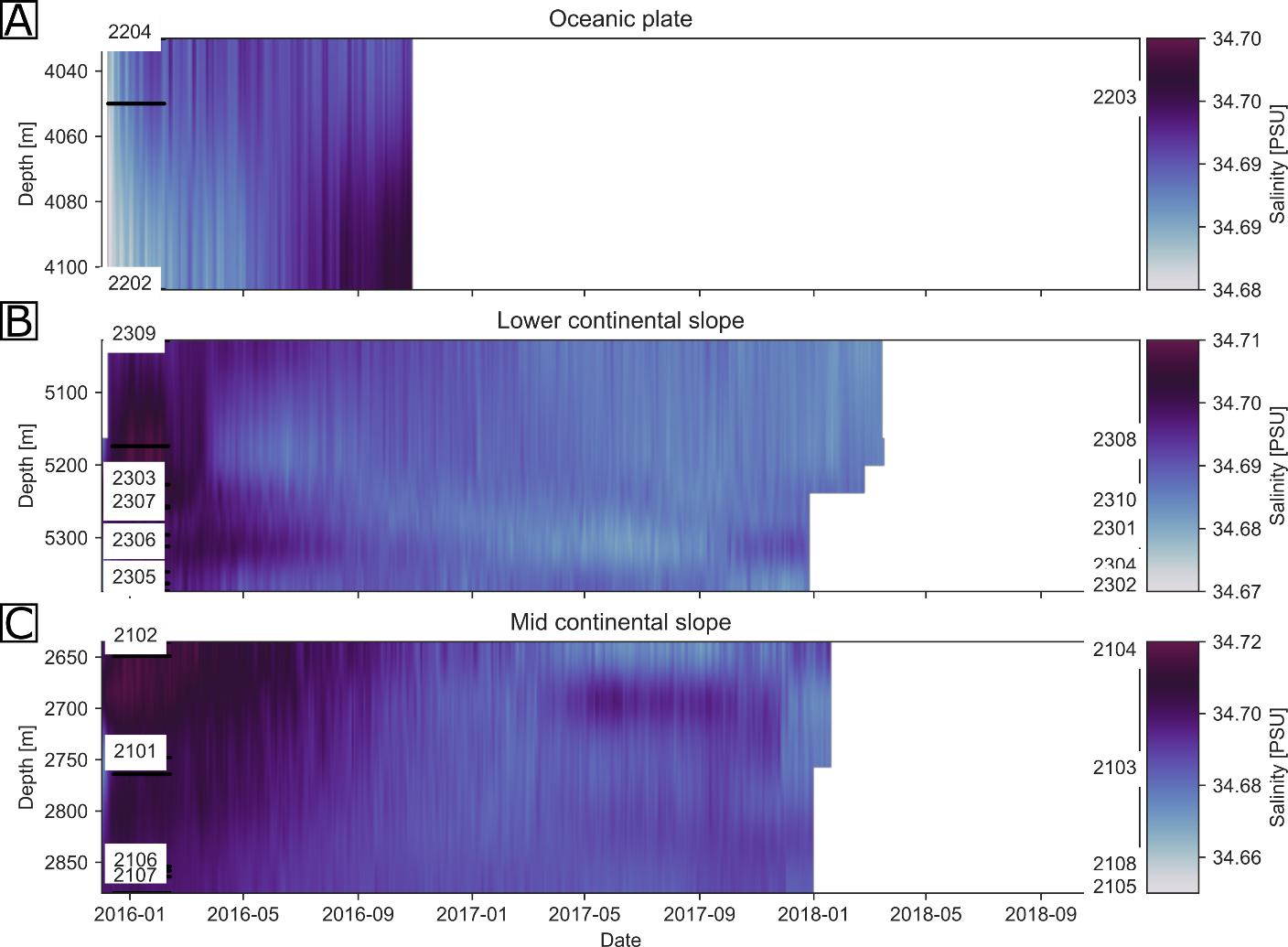


Figure S7.

Salinity depth section of the three seafloor array sites (Panels A-C). The salinity was derived from sound speed measurements and was gridded day wise with a vertical resolution of 40m.

**REFERENCES AND NOTES**

1. Lange, D. *et al.* Interseismic strain build-up on the submarine North Anatolian Fault offshore Istanbul. *Nat Commun* **10**, 3006 (2019).

2. Petersen, F., Kopp, H., Lange, D., Hannemann, K. & Urlaub, M. Measuring tectonic seafloor deformation and strain-build up with acoustic direct-path ranging. *Journal of Geodynamics* **124**, 14–24 (2019).

3. Urlaub, M. *et al.* Gravitational collapse of Mount Etna’s southeastern flank. *Science Advances* **4**, eaat9700 (2018).

4. Watts, D. R. & Kontoyiannis, H. Deep-Ocean Bottom Pressure Measurement: Drift Removal and Performance. *Journal of Atmospheric and Oceanic Technology* **7**, 296–306 (1990).
